# A systematic synthesis of direct costs to treat and manage tuberculosis disease applied to California, 2015

**DOI:** 10.1186/s13104-017-2754-y

**Published:** 2017-08-30

**Authors:** Peter Oh, Lisa Pascopella, Pennan M. Barry, Jennifer M. Flood

**Affiliations:** California Department of Public Health, Center for Infectious Diseases, Tuberculosis Control Branch, 850 Marina Bay Parkway P2, Richmond, CA 94804 USA

## Abstract

**Background:**

The cost of treating and managing cases of active tuberculosis (TB) disease—from diagnosis to treatment completion—is needed by agencies working on public health budgets, resource allocation and cost-effectiveness analysis. Although components of TB costs have been published in the United States (US), no recent study has assessed overall costs for TB care and potential gaps. To systematically review the US literature for costs of treating and managing cases of active TB disease, adjust these costs to current (2015) values, and assess gaps. We quantified total direct costs—from the perspective of the health care payer—of the treatment and case management of active TB disease. Estimates were based on published figures in the US, and operational data of the California Department of Public Health.

**Result:**

The average direct cost of treating and managing a TB case was $34,600 in 2015. The average cost of a multidrug-resistant TB case was $110,900. Health care spending for treating and case managing TB patients in California amounted to approximately $75.6 million for the 2133 new cases reported in 2015. Most published cost estimates were based on data from the 1990s.

**Conclusion:**

TB is resource-intensive to treat and manage. Our synthesis provides inputs for budgets and economic analyses. New studies to provide original cost data are needed to better reflect current clinical and public health practices.

**Electronic supplementary material:**

The online version of this article (doi:10.1186/s13104-017-2754-y) contains supplementary material, which is available to authorized users.

## Background

Tuberculosis (TB) remains a substantial public health challenge in the United States (US), with 9563 new cases of the disease reported in 2015 [1]. The costs of treating and managing tuberculosis are key inputs for economic evaluations to assess the cost-effectiveness of public health interventions. Health policy-making, public health department budget planning, and advocacy efforts also benefit from accurate estimates of TB costs, but recently published information on their magnitude in the US setting is limited. We reviewed the literature for original US-based data on the direct health care costs of treating and managing TB disease, and adjusted costs to 2015 US dollars. Our main objective was to generate comprehensive, literature-based, inflation-adjusted medical cost estimates. A secondary objective was to qualitatively assess gaps in the current knowledge of TB costs, to guide future research on the topic.

## Methods

We performed a literature review, identified and adjusted the average costs of TB reported in the literature, and described gaps and trends in reported costs. We used an ingredients framework, in which the total cost of a program or intervention is estimated by summing each component of cost [2]. The six direct cost components in our analysis were (1) hospitalization, (2) inpatient physician services, (3) outpatient physician services, (4) outpatient case management (clinic visits and case management personnel time for directly observed therapy), (5) laboratory and imaging tests, and (6) anti-TB medications. First, we reviewed the literature for published original cost estimates for any of these components. We filled gaps in the literature (i.e., a component for which we found no published cost) by using federal Medicare data to calculate estimates based on service utilization characteristics of an average case, according to TB surveillance and operational data in California. We adjusted all estimates for inflation to reflect prices in 2015 using specific components of the Consumer Price Index (CPI) as detailed in the online appendix (Additional file 1). Finally, we stratified our analysis by multidrug-resistant TB (MDR TB resistance to at least isoniazid and rifampin) and drug-susceptible TB because MDR TB is substantially more costly than drug-susceptible TB.

We searched the Scopus electronic database in January 2016 for articles published between 1990 and 2015 that reported original data on direct costs of tuberculosis treatment and case management. We used the search term “tuberculosis cost” and limited results to articles in the life sciences, health sciences, and social sciences and humanities. We limited the search to English-language articles, and US-based costs. We chose the narrow focus of US-based costs because the health care system in the US is different than that in other low TB incidence/high income countries (e.g. Western European nations). Second, two authors (PO, LP) reviewed the titles and abstracts of the articles and excluded those in which the term cost was used in terms other than financial cost (e.g., fitness costs to the *Mycobacterium tuberculosis* organism due to genetic mutations), and TB disease in animals. The same authors reviewed the remaining articles in full to identify those with original estimates of direct costs of any TB cost component (as opposed to secondary cost estimates citing other work). The perspective of our analysis was the health care payer of medical services. We limited our estimates to the costs of treatment and case management of active TB cases, and did not include any of the following: indirect and societal costs (e.g., productivity losses incurred by TB patients); costs of the workup leading to diagnosis of TB; or costs of contact investigation. We also excluded estimates related to inpatient care that were based on charge data, because hospital charges are considered unreliable proxies for costs.

If an article reported the cost of an episode of a component, we adjusted this cost to a per-patient basis by multiplying it by the average utilization, based on surveillance data in the California Department of Public Health (CDPH) TB registry [3], and CDPH operational databases. The online appendix provides further detail about the data sources (Additional file 2).

If the literature search did not yield a published estimate for a component of TB care, we calculated a value based on published federal government reimbursement rates for clinical services comprising the component, multiplied by the utilization for an average TB case patient in California. The total cost per active TB case was estimated using mean values for the length of treatment, number of clinic visits, complexity of clinic visits, and type and frequency of laboratory and chest radiograph exam. Details of these parameters and inputs are described in the online appendix (Additional file 3).

## Results

**Fig. 1:**
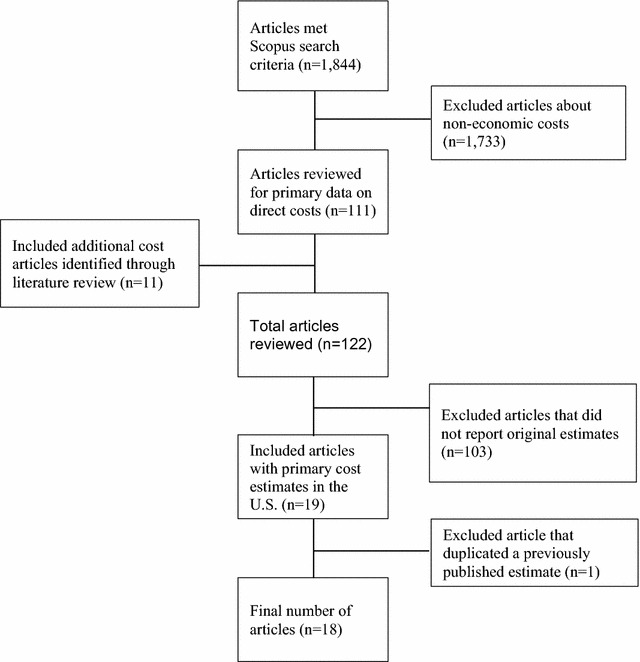
Literature review and selection of articles reporting primary TB cost data in the US, 1990–January 2016

The figure diagrams the literature search and the process by which the articles were selected. The Scopus search term combination and inclusion criteria yielded 1844 articles. The abstracts of 111 papers suggested content on TB costs in the US, and these articles were reviewed in full. Examination of these articles and their references led to the identification of eleven additional relevant articles. Of the 122 articles reviewed in full, 19 reported primary data on direct costs of TB treatment and case management. The other articles presented secondary costs based on previously published estimates, or used charges instead of costs, and were therefore excluded from our analysis. One of the 19 articles with primary data used identical estimates presented in a previous article by the same authors and was excluded to avoid duplication, yielding 18 articles meeting all inclusion criteria (Fig. 1).

The inflation-adjusted per-patient direct cost estimates calculated from the values reported in these 18 articles are summarized in Table 1. We identified five articles that reported costs of hospitalization of drug-susceptible TB, and one additional article for MDR TB. Two of the TB estimates were reported as per-episode costs, which we adjusted to average per-patient values. The per-patient cost of hospitalization of drug-susceptible cases ranged from $10,100 to $45,400, with an average of $24,000. The single article with original cost data on MDR TB inpatient care was determined from data in 1995/96, an estimated $43,300. Five of the six hospitalization estimates were based on data from the mid-1990s or earlier, with only one estimate based on observations in the 2000s. Inpatient physician fees for TB were reported in two articles as $3400 and $3800, for an average of $3600 per TB case-patient. One article reported physician inpatient fees for MDR TB at $4000. All of the published physician fee estimates stemmed from the early and mid-1990s. Outpatient physician fees for TB were reported in four articles and averaged $400 per TB case (range $200–$600). The most recent of these articles had the lowest cost estimate [4]. One additional article, using data from 1995, estimated outpatient physician fees for MDR TB at $1200 per patient [5]. The outpatient case management cost of TB ranged from $1400 to $8000 in the five articles reporting estimates on this component, with an average of $4300. Two of these articles used data from the early 1990s and three on data from the 2000s. For MDR TB, outpatient case management costs were reported in two studies using data from the 1990s, with an average value of $27,800 (range $21,500–$34,100).

**Table 1:** Direct costs per TB case in the US, by cost component, adjusted to 2015 dollars

Ref.	Type of TB	Setting, US state(s)	Sample year(s)	Sample size	Hospitalization	Physician fees, inpatient	Physician fees, outpatient	Outpatient case management	Laboratory and imaging tests	Medications
[20]	Drug-susceptible	16 states^a^	1990	n/s	26,900^b^	3800		6200		
	MDR							21,500		
[21]	Drug-susceptible	IA	1991	n/a	10,100		600		2200	800
[14]	Drug-susceptible	CA, MS, NJ	1992	178					1600	1200
[22]	Drug-susceptible	PA	1992	18	45,400					
[23]	Drug-susceptible	MD	1992–94	n/s			600	3100	1200	1300
[24]	Drug-susceptible	MA	1993	n/a						1300
	MDR									16,900
[25]	Drug-susceptible	IL	1993–94	90		3400			1300	
[5]	Drug-susceptible	CA	1995				300			
	MDR	CA	1995				1200			
[10]	Drug-susceptible	7 states^c^	1995–96	733	19,300					
[26]	MDR	7 states^c^	1995–96	13	43,300	4000		34,100		
[8]	Drug-susceptible	US	2006	8800	18,300^b^					
[27]	Drug-susceptible	OR	2006	42				8000		
[28]	MDR	CA, NY, TX	2005–07	130						62,600
[6]	Drug-susceptible	NC	2008–10	99				1400	1400	300
[29]	MDR	US	2009	n/a						11,600
[30]	Drug-susceptible	TN	2010	n/a				2900		500
[4]	Drug-susceptible	US	2011	n/a			200			
[31]	Drug-susceptible	US	2014	n/a						300

*n/s* not specified, *n/a* not applicable (i.e., estimates not based on a sample of cases, but on administrative data)

^a^AZ, CA, CO, FL, MA, ME, NH, NJ, NV, NY, OR, PA, SC, VT, WA, WI

^b^The reported value was per hospitalization episode, and was adjusted to a per-case patient estimate using the utilization assumptions detailed in Additional file 3

^c^CA, GA, IL, MS, NY, SC, TX

Laboratory and imaging test costs for TB were reported in five studies, with an average value of $1500. One of these studies used data as recent as 2010 [6], whereas the others were based on data in the early 1990s. We found no reported costs of laboratory and imaging tests for MDR TB treatment. We estimated this component to be $4200, based on the schedule of tests recommended by the California MDR TB Service and Medicare Clinical Laboratory Fee Schedule.

Anti-TB medication cost data were reported in seven articles, with a range from $300 to $1300 and an average of $800. Four of the estimates were taken in the early 1990s, and three between 2008 and 2014. Medication costs for MDR TB were found in three studies, with a range of $11,600 to $62,600 and average of $30,400.

The sum of the average cost components for treatment and case management was $34,600 per patient for drug-susceptible TB and $110,900 for MDR TB. Applying these cost estimates to the number of new cases of TB and MDR TB reported in California, approximately $75.6 million was spent by the public health programs to treat and manage the 2133 case patients diagnosed in 2015 (Table 2). Hospitalization was the largest cost component for TB (69%) and MDR TB (39%). Medications represented 2% of direct cost of treating TB but 27% of the costs of MDR TB. MDR TB cases incurred a disproportionate share of the total direct costs of TB (1% of the case burden and 3% of costs).

**Table 2:** Average per-patient cost estimates and statewide direct health care costs, by component, California, 2015

Cost component	Per non-MDR TB patient Average (range of averages)	Per MDR TB patient Average (range of averages)	All non-MDR TB cases in CA, 2015 N = 2110 $ average (range of averages)	All MDR TB cases in CA, 2015 N = 23 $ average (range of averages)
Hospitalization	24,000 (10,100–45,400)	43,300	50,640,000 (21,311,000–95,794,000)	995,900 (*)
Inpatient physician fees	3600 (3400–3800)	4000	7596,000 (7174,000–8018,000)	92,000 (*)
Outpatient physician fees	400 (200–600)	1200	844,000 (422,000–1266,000)	27,600 (*)
Case management	4300 (1400–8000)	27,800 (21,500–34,100)	9073,000 (2954,000–16,880,000)	639,400 (494,500–784,300)
Laboratory and imaging tests	1500 (1200–2200)	4200	3165,000 (2532,000–4642,000)	96,600 (*)
Anti-TB medications	800 (300–1300)	30,400 (11,600–62,600)	1688,000 (633,000–2745,500)	699,200 (266,800–1439,800)
Subtotal	34,600 (16,600–61,300)	110,900 (85,800–149,400)	73,006,000 (35,026,000–129,343,000)	2550,700 (1973,400–3436,200)
Total direct medical and public health cost of TB disease in California, 2015			75,556,700 (36,999,400–132,779,200)	

*No range of average shown because only one estimate met the literature search inclusion criteria

## Discussion

To our knowledge, this is the first published synthesis of the total direct costs of the treatment and management of active TB disease in the US. Based on inflation-adjusted estimates reported in the literature, the direct cost of treating and managing the care of one TB case patient was, on average, $34,600 in 2015 dollars. At $110,900, the average health care cost of an MDR TB case was three times higher than the average cost for a drug-susceptible case, even though the average costs of medication for MDR-TB were 38 times higher than those for drug-susceptible TB. Applying these average values to the number of new TB cases reported in 2015, we estimate that the average health care spending to treat and manage new cases of TB in California totals $75.6 million per year, with the range of averages spanning $37–$133 million per year. We believe these estimates, based on older published literature, are an underestimate of medical and public health costs of TB in the US and we further describe our reasoning below.

Even so, these estimated medical costs of treatment and case management are substantial, and represent a sizeable portion of the financial resources expended to combat TB. In California, funding for TB control and prevention is a blend of county and state budgets, as well as federal funding from the Centers for Disease Control and Prevention (CDC). In 2010, CDC funded California at a level of $17.6 million [7]. These dollars are directed largely toward efforts such as diagnosing cases of active TB disease and ensuring completion of therapy, contact investigation, surveillance and laboratory activities—the essential services that are traditionally associated with public health agencies. While the majority of this funding supports population-based services that fall outside the scope of the medical care system, a small portion may support the clinical services vital to arresting the spread of TB. Our analysis shows that just the medical costs of treatment and case management—of a preventable disease that barely registers in the public consciousness—are tens of millions of dollars, and far exceed the annual federal funding awards aimed at controlling TB. Our cost estimates would be substantially larger, had we included additional costs such as personal productivity losses, disability and mortality. From the broader, societal perspective of costs incurred by TB disease, our literature-based calculations could serve as minimal estimates of future expenditures that could be saved by more effective, targeted screening programs aimed at preventing TB disease.

Our systematic review identified several gaps in the literature on direct costs of TB treatment. Only 8 of the 18 articles reviewed in our study were based on data since 2000. For several cost components, original data have not been reported since the mid-1990s (e.g., physician fees). Hospitalization costs for TB (not specific to MDR TB) were the most frequently reported component and the largest single contributor of the total direct cost of TB care, but the most recent of these estimates was based on 2006 data [8]. The results of our review suggest a need for updated estimates of the costs of TB hospitalization because TB hospitalization practices may have changed since then. A study of human immunodeficiency virus (HIV) testing of TB case patients in California in 2008 suggested that as few as one-third of TB patients were hospitalized [9], compared to one half of patients in the mid-1990s [10]. On the other hand, a study of 135 MDR TB patients in the US during 2005–2007 demonstrated longer times in the hospital, and increased drug resistance, which cost more, compared to study cohorts from a 1996 study [11].

Our study had several limitations. Given our focus on indexed medical and public health literature, we may have missed cost estimates reported in the gray literature (e.g., program reports by governmental agencies, policy briefs by nonprofit organizations). Cost-effectiveness research norms generally encourage the adoption of a societal perspective of health care costs, such that all costs (and effects) are measured, including those affecting the patient (e.g., out-of-pocket costs, lost economic productivity) [12]. The perspective of our synthesis—the health care payer—is more restricted than the societal perspective, but has the potential advantage of being a more pragmatic perspective for policy-makers focused on assessing the impact of changing expenditures to prevent TB.

Important public health interventions to combat TB in the US such as contact investigation were not included in our estimates. Evaluating persons with suspected TB also contributes to underestimation of TB costs [13] and are also not counted in our estimates. Furthermore, we did not consider the excess costs associated with TB patients lost to follow-up. Such patients could incur additional medical and public health expenditures due to relapse of disease, infection of additional contacts, and the development of drug resistance [14]. Due to data limitations we could not stratify our cost analysis by public versus private sector, which may be an important distinction. The impact of the changing health care system on the costs of TB care may be substantial, as some care traditionally provided by public health clinics shifts to primary care and community clinics [15]. Finally, this analysis did not take into account upward pressures on costs such as anti-TB drug shortages in the US [16]. Shortages force public health clinics and other TB care providers to spend more on alternative anti-TB drugs and increased monitoring for side effects. Mathematical models of the impact of drug shortages suggest a several-fold increase in the medication costs borne by public health programs [17]. Shortages and sudden dramatic price increases of second-line drugs that are needed to treat MDR TB have become more common in recent years [18, 19] and could have led to underestimates of the medications cost component.

These limitations underscore the likelihood that reported costs substantially underestimate the true direct costs of TB, especially for complicated cases such as extensively drug-resistant (XDR) TB, for which treatment costs likely far exceed our conservative estimates [11]. Nevertheless, our review offers several strengths. We observed that it was not uncommon for cost studies to cite intermediary articles as sources instead of the original source, thereby creating the misleading perception that primary TB cost data sources in the literature are more numerous and more current than they actually are. By distilling the TB cost literature down to only the original data sources and their publication years, we offer a practical resource for economic analysts to assess the applicability of older cost estimates in the context of the TB control interventions and practices in place today.

## Conclusion

TB is resource-intensive to treat and manage. Our synthesis provides inputs for budgets and economic analyses, based on currently available data on TB costs. New studies to provide original cost data are needed to better reflect current clinical and public health practices.

## Additional files

**Additional file 1.** Consumer Price Index (CPI) components used in the analysis.

**Additional file 2.** Operational databases used in the analysis.

**Additional file 3.** Input parameters and values used in calculations of cost adjustments (from per-episode to per-patient) and cost estimates for components not reported in the literature.

## Abbreviations

TB: tuberculosis
MDR: multidrug-resistant (i.e. resistant to at least isoniazid and rifampin)
U.S.: United States
CDPH: California Department of Public Health
CDC: Centers for Disease Control and Prevention
HIV: human immunodeficiency virus
XDR: extensively drug-resistant (i.e. MDR and resistant to a fluoroquinolone and a second-line injectable anti-TB drug)
