# Supplementary material for: A systematic synthesis of direct costs to treat and manage tuberculosis disease applied to California, 2015
Source: BMC Res Notes. 2017 Aug 30;10:434. doi: 10.1186/s13104-017-2754-y (PMC5577675; doi:10.1186/s13104-017-2754-y)
Supplement: Supplementary file 1 — Additional file 1. Consumer Price Index (CPI) components used in the analysis. [file 13104_2017_2754_MOESM1_ESM.docx]

Additional file 1. Consumer Price Index (CPI) components used in the analysis

| Cost component | CPI component |
| --- | --- |
| Hospitalization | Hospital and related services |
| Inpatient physician services | Physician services |
| Outpatient physician services | Physician services |
| Outpatient case management | Medical care services |
| Laboratory and imaging tests | Medical care services |
| Anti-TB medications | Prescription drugs |

Note: The geographic area used for all CPI components was “U.S. city average”
