# Supplementary material for: A systematic synthesis of direct costs to treat and manage tuberculosis disease applied to California, 2015
Source: BMC Res Notes. 2017 Aug 30;10:434. doi: 10.1186/s13104-017-2754-y (PMC5577675; doi:10.1186/s13104-017-2754-y)
Supplement: Supplementary file 2 — Additional file 2. Operational databases used in the analysis. [file 13104_2017_2754_MOESM2_ESM.docx]

Additional file 2. Operational databases used in the analysis

The first operational data source consisted of administrative records of the MDR/XDR (extensively drug resistant) TB Enhanced Surveillance (MTES) project. MTES was a joint effort of the California Department of Public Health (CDPH) TB Control Branch and the Centers for Disease Control and Prevention Division of TB Elimination implemented between 2005 and 2007 to prospectively collect data on diagnostic and case management factors to improve early detection of MDR TB cases. We queried this operational dataset for average values of MDR TB treatment duration, and the frequency of hospitalization. The second source of operational data was the CDPH MDR TB Service (MDR Service), an expert consultancy that provides clinical and case management guidance to local health department TB control agencies in California treating patients with MDR TB. We used MDR Service-recommended schedules of clinical tests and procedures for MDR TB case management and the Centers for Medicare & Medicaid Services Clinical Laboratory Fee Schedule to estimate the cost of the “laboratory and imaging tests” component.
