# Supplementary material for: A systematic synthesis of direct costs to treat and manage tuberculosis disease applied to California, 2015
Source: BMC Res Notes. 2017 Aug 30;10:434. doi: 10.1186/s13104-017-2754-y (PMC5577675; doi:10.1186/s13104-017-2754-y)
Supplement: Supplementary file 3 — Additional file 3. Input parameters and values used in calculations of cost adjustments (from per-episode to per-patient) and cost estimates for components not reported in the literature. [file 13104_2017_2754_MOESM3_ESM.docx]

Additional file 3. Input parameters and values used in calculations of cost adjustments (from per-episode to per-patient) and cost estimates for components not reported in the literature.

| Component | Parameter | Value | Reference or source |
| --- | --- | --- | --- |
| Hospitalization, TB | Proportion of TB case patients hospitalized | 50% | Ref. 10 |
|  | Average number of hospitalizations per hospitalized TB case | 1.12 | Ref. 10 |
| Hospitalization, MDR TB | Proportion of MDR TB case hospitalized | 75% | California MDR/XDR TB Enhanced Surveillance operational database |
| Laboratory and imaging tests, MDR TB | Number of AFB smear and  culture specimens processed | 28 | MDR Service recommendation |
|  | Number of chest radiographs | 7 | MDR Service recommendation |
|  | Number of drug susceptibility tests | 1.3 | MDR Service recommendation, CA surveillance data |
